# Poly[[tetra­aqua­bis[μ_4_-2,2′-(*p*-phenyl­ene­di­oxy)diacetato][μ_2_-2,2′-(*p*-phenyl­ene­di­oxy)diacetato]dierbium(III)] hexa­hydrate]

**DOI:** 10.1107/S1600536809046613

**Published:** 2009-11-11

**Authors:** Dan-Yi Wei, Yan-Guang Zhang, Mei-Li Wang, Zhen-Ke Zhu

**Affiliations:** a State Key Laboratory Base of Novel Functional Materials and Preparation Science, Faculty of Materials Science and Chemical Engineering, Ningbo University, Ningbo, Zhejiang 315211, People’s Republic of China

## Abstract

The asymmetric unit of the title compound, [Er_2_(C_10_H_8_O_6_)_3_(H_2_O)_4_]·6H_2_O, comprises one Er^3+^ ion, one and a half 2,2′-(*p*-phenyl­enedi­oxy)diacetate (hqda) ligands, two coordinated water mol­ecules and three uncoordinated water mol­ecules. The Er^3+^ ion is nine-coordinated by seven O atoms from hqda ligands and two O atoms from water mol­ecules. In the title compound, there are two types of crystallographically independent ligands: one with an inversion center in the middle of the ligand is chelating on both ends of the ligand towards each one Er center; the other hqda ligands are bridging-chelating on one side, and bridging on the other end of the ligand. Two adjacent Er^3+^ ions are thus chelated and bridged by –COO groups from hqda ligands in three coordination modes (briding–chelating, bridging and chelating). These building blocks are linked by OOC—CH_2_O—C_6_H_4_—OCH_2_—COO spacers, forming two-dimensional neutral layers. Adjacent layers are linked by O—H⋯O hydrogen-bonding inter­actions, forming a three-dimensional supermolecular network.

## Related literature

For general background to metal-organic frameworks, see: Maji *et al.* (2005 ▶); Moulton & Zaworotko (2001 ▶); Rao *et al.* (2004 ▶); Sun *et al.* (2006 ▶); Zou *et al.* (2006 ▶); Burrows *et al.* (2000 ▶); Huang *et al.* (2005 ▶). For related stuctures, see: Hong *et al.* (2006 ▶); Li *et al.* (2008 ▶).
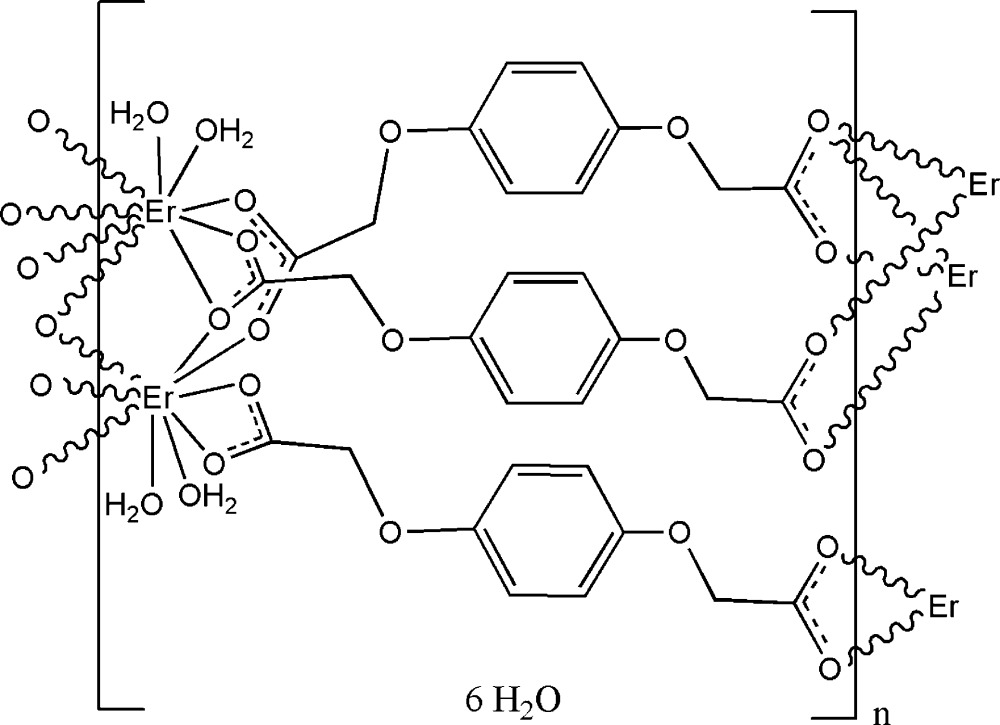


## Experimental

### 

#### Crystal data

[Er_2_(C_10_H_8_O_6_)_3_(H_2_O)_4_]·6H_2_O
*M* *_r_* = 1187.17
Triclinic, 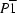

*a* = 8.5993 (17) Å
*b* = 9.6356 (19) Å
*c* = 12.689 (3) Å
α = 102.46 (3)°
β = 95.28 (3)°
γ = 106.69 (3)°
*V* = 970.0 (4) Å^3^
*Z* = 1
Mo *K*α radiation
μ = 4.40 mm^−1^
*T* = 298 K
0.43 × 0.29 × 0.15 mm

#### Data collection

Rigaku R-AXIS RAPID diffractometer
Absorption correction: multi-scan (*ABSCOR*; Higashi, 1995 ▶) *T*_min_ = 0.312, *T*_max_ = 0.535
9659 measured reflections
4403 independent reflections
4219 reflections with *I* > 2σ(*I*)
*R*_int_ = 0.030

#### Refinement

*R*[*F* ^2^ > 2σ(*F* ^2^)] = 0.019
*wR*(*F* ^2^) = 0.044
*S* = 1.17
4403 reflections
271 parameters
1 restraint
H-atom parameters constrained
Δρ_max_ = 0.46 e Å^−3^
Δρ_min_ = −1.08 e Å^−3^

### 

Data collection: *RAPID-AUTO* (Rigaku, 1998 ▶); cell refinement: *RAPID-AUTO*; data reduction: *CrystalStructure* (Rigaku/MSC, 2002 ▶); program(s) used to solve structure: *SHELXS97* (Sheldrick, 2008 ▶); program(s) used to refine structure: *SHELXL97* (Sheldrick, 2008 ▶); molecular graphics: *ORTEPII* (Johnson, 1976 ▶); software used to prepare material for publication: *SHELXL97*.

## Supplementary Material

Crystal structure: contains datablocks global, I. DOI: 10.1107/S1600536809046613/zl2248sup1.cif

Structure factors: contains datablocks I. DOI: 10.1107/S1600536809046613/zl2248Isup2.hkl

Additional supplementary materials:  crystallographic information; 3D view; checkCIF report

## Figures and Tables

**Table 1:** Hydrogen-bond geometry (Å, °)

*D*—H⋯*A*	*D*—H	H⋯*A*	*D*⋯*A*	*D*—H⋯*A*
O10—H10*D*⋯O7^i^	0.82	2.09	2.880 (4)	163
O10—H10*C*⋯O12	0.82	1.97	2.732 (4)	154
O11—H11*D*⋯O13	0.82	1.82	2.634 (4)	173
O11—H11*C*⋯O12	0.82	1.92	2.709 (4)	161
O12—H12*D*⋯O8^ii^	0.82	2.00	2.798 (4)	167
O12—H12*C*⋯O14^iii^	0.82	1.97	2.780 (4)	172
O13—H13*D*⋯O7^iv^	0.82	2.12	2.872 (4)	151
O13—H13*C*⋯O3	0.82	2.03	2.804 (4)	157
O14—H14*C*⋯O6	0.82	2.17	2.874 (4)	144

Symmetry codes: (i) 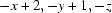; (ii) 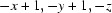; (iii) 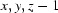; (iv) 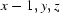.

**Fig. 1.**
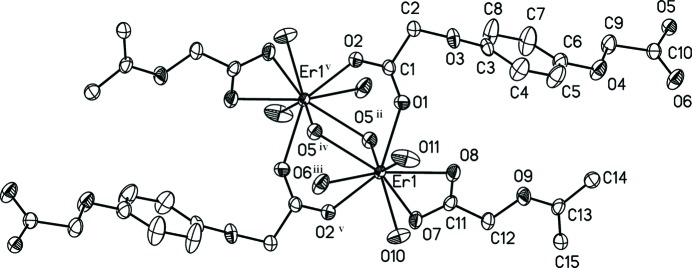
ORTEP view of complex molecule of the title compound. Displacement ellipsoids are drawn at the 45% probability level. H atoms and lattice water molecules were omitted for clarity. (Symmetry codes: ii = -x + 1, -y + 1, -z; iii = x, y, z-1; iv = x - 1, y, z; v = -x + 2, -y + 2, -z).

**Fig. 2.**
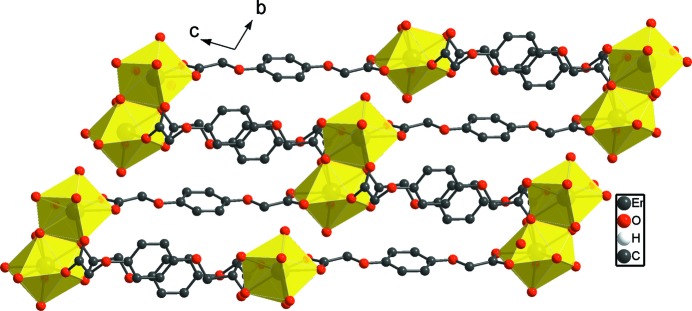
Two-dimensional layer in the title compond perpendicular to the [100] direction with H-atoms and lattice water molecules omitted.

## supplementary crystallographic information

### Comment

The investigation of the assembly of metal-organic frameworks (MOFs) has attracted great interest due to their versatile architecture and promising applications for ion exchange, gas storage, separation, and catalysis (Maji *et al.*, 2005; Moulton & Zaworotko 2001; Rao *et al.*, 2004; Sun *et al.*, 2006; Zou *et al.*, 2006). The selection of multifunctional bridging ligands is crucial to synthesize novel MOFS (Burrows *et al.*, 2000; Huang *et al.*, 2005). Among these, hydroquinone-*O,O*'-diacetic acid (H_2_hqda) is a good ligand in the preparation various metal-organic coordination polymers. Recently, several lanthanide(III) hqda compounds with fascinating structures have been reported (Hong *et al.*, 2006; Li *et al.*, 2008). Herein, we report a new compound [Er_2_(hqda)_3_(H_2_O)_4_].6H_2_O.

The asymmetric unit of the title compound comprises one Er^3+^ ion, one and a half 2,2'-(*p*-phenylenedioxy)diacetate anions (hqda), two coordinated water molecules and three lattice water molecules. The Er^3+^ ion is nine coordinated by seven oxygen atoms of hqda ligands and two oxygen atoms of aqua ligands (Fig 1). The Er—O (carboxylate) distances fall in the range 2.341 (2)–2.529 (2) Å, and those of the Er—O (water) bonds are 2.317 (2) Å and 2.368 (2) Å, respectively. The coordination environment of the Er^3+^ ion may be described as a distorted tricapped trigonal prism. In the title compound, there are two types of crystallographically independent ligands. One type with an inversion center in the middle of the ligand is chelating on both ends of the ligand towards each one Er center. The other type is bridging-chelating on one side, and bridging on the other, thus connecting each two Er centers with each other. Two adjacent Er^3+^ ions are thus chelated and briged by –COO groups from hqda ligands in three coordination modes (briding-chelating, bridging and chelating modes) to form [Er_2_(hqda)_3_(H_2_O)_4_].6H_2_O building blocks. These building blocks are linked by the OOC–CH_2_O–C_6_H_4_–OCH_2_COO (hqda) spacers to form two-dimensional neutral layers perpendicular to the [100] direction (Fig 2). The lattice water molecules are sandwiched between these two-dimensional layers and hydrogen bonded with them. The adjacent two-dimensional layers are further interlinked by these hydrogen bonds to form a three-dimensional supermolecular network.

### Experimental

All commercially available chemicals were of reagent grade and used without further purification. Er(NO_3_)_3_.6H_2_O (0.0922 g, 0.2 mmol) and H_2_hqda (0.0452 g, 0.2 mmol) were added to a stirred solution of 20 ml dimethyl formamide/H_2_O to form a clear solution, which was mixed with 5 ml ethanol and 0.15 ml triethylamine. The resulting solution was kept at room temperature and pink, block-like crystals grew after ca. 20 days.

### Refinement

H atoms bonded to C atoms were placed in geometrically calculated positons and refined using a riding moldel, with distances of C—H = 0.93Å (benzene ring) and 0.97Å (–CH_2_), and *U*_iso_(H) = 1.2U_eq_(C). Water H atoms were positioned geometrically and refined with distance restraints of O—H = 0.82 (2) Å and *U*_iso_(H) = 1.5U_eq_(O).

### Crystal data

**Table:**

[Er_2_(C_10_H_8_O_6_)_3_(H_2_O)_4_]·6H_2_O	*Z* = 1
*M**_r_* = 1187.17	*F*(000) = 584
Triclinic, *P*1	*D*_x_ = 2.032 Mg m^−^^3^
Hall symbol: -P 1	Mo *K*α radiation, λ = 0.71073 Å
*a* = 8.5993 (17) Å	Cell parameters from 8985 reflections
*b* = 9.6356 (19) Å	θ = 3.2–27.5°
*c* = 12.689 (3) Å	µ = 4.40 mm^−^^1^
α = 102.46 (3)°	*T* = 298 K
β = 95.28 (3)°	Block, pink
γ = 106.69 (3)°	0.43 × 0.29 × 0.15 mm
*V* = 970.0 (4) Å^3^	

### Data collection

**Table:**

Rigaku R-AXIS RAPID diffractometer	4403 independent reflections
Radiation source: fine-focus sealed tube	4219 reflections with *I* > 2σ(*I*)
graphite	*R*_int_ = 0.030
Detector resolution: 0 pixels mm^-1^	θ_max_ = 27.5°, θ_min_ = 3.2°
ω scans	*h* = −9→11
Absorption correction: multi-scan (*ABSCOR*; Higashi, 1995)	*k* = −12→12
*T*_min_ = 0.312, *T*_max_ = 0.535	*l* = −16→16
9659 measured reflections	

### Refinement

**Table:**

Refinement on *F*^2^	Primary atom site location: structure-invariant direct methods
Least-squares matrix: full	Secondary atom site location: difference Fourier map
*R*[*F*^2^ > 2σ(*F*^2^)] = 0.019	Hydrogen site location: inferred from neighbouring sites
*wR*(*F*^2^) = 0.044	H-atom parameters constrained
*S* = 1.17	*w* = 1/[σ^2^(*F*_o_^2^) + (0.0129*P*)^2^ + 0.5344*P*] where *P* = (*F*_o_^2^ + 2*F*_c_^2^)/3
4403 reflections	(Δ/σ)_max_ = 0.001
271 parameters	Δρ_max_ = 0.46 e Å^−^^3^
1 restraint	Δρ_min_ = −1.08 e Å^−^^3^

### Special details

**Table:**

Geometry. All e.s.d.'s (except the e.s.d. in the dihedral angle between two l.s. planes) are estimated using the full covariance matrix. The cell e.s.d.'s are taken into account individually in the estimation of e.s.d.'s in distances, angles and torsion angles; correlations between e.s.d.'s in cell parameters are only used when they are defined by crystal symmetry. An approximate (isotropic) treatment of cell e.s.d.'s is used for estimating e.s.d.'s involving l.s. planes.
Refinement. Refinement of *F*^2^ against ALL reflections. The weighted *R*-factor *wR* and goodness of fit *S* are based on *F*^2^, conventional *R*-factors *R* are based on *F*, with *F* set to zero for negative *F*^2^. The threshold expression of *F*^2^ > σ(*F*^2^) is used only for calculating *R*-factors(gt) *etc*. and is not relevant to the choice of reflections for refinement. *R*-factors based on *F*^2^ are statistically about twice as large as those based on *F*, and *R*- factors based on ALL data will be even larger.

### Fractional atomic coordinates and isotropic or equivalent isotropic displacement parameters (Å^2^)

**Table:**

	*x*	*y*	*z*	*U*_iso_*/*U*_eq_	
Er1	0.869905 (13)	0.797688 (12)	−0.007667 (8)	0.01362 (4)	
O1	0.7733 (2)	0.9698 (2)	0.10577 (15)	0.0238 (4)	
O2	0.9134 (2)	1.2049 (2)	0.10636 (14)	0.0220 (4)	
O3	0.5844 (2)	1.0620 (2)	0.24732 (14)	0.0230 (4)	
O4	0.7484 (3)	0.8777 (2)	0.61490 (15)	0.0307 (5)	
O5	0.8979 (2)	1.0035 (2)	0.90125 (13)	0.0189 (4)	
O6	0.7683 (3)	0.7795 (2)	0.79481 (15)	0.0295 (5)	
O7	1.0417 (2)	0.6690 (2)	0.07609 (14)	0.0224 (4)	
O8	0.8399 (2)	0.7140 (2)	0.15645 (15)	0.0239 (4)	
O9	0.9096 (3)	0.5949 (3)	0.32042 (15)	0.0267 (5)	
O10	0.7747 (2)	0.5361 (2)	−0.08718 (17)	0.0293 (5)	
H10D	0.8146	0.4726	−0.0746	0.044*	
H10C	0.6899	0.4916	−0.1317	0.044*	
O11	0.5849 (3)	0.7150 (3)	−0.03432 (19)	0.0386 (6)	
H11D	0.5209	0.7517	−0.0033	0.058*	
H11C	0.5269	0.6420	−0.0823	0.058*	
C1	0.8034 (3)	1.1075 (3)	0.13378 (19)	0.0173 (5)	
C2	0.6953 (4)	1.1713 (3)	0.2068 (2)	0.0235 (6)	
H2B	0.7656	1.2496	0.2684	0.028*	
H2A	0.6324	1.2167	0.1655	0.028*	
C3	0.6421 (3)	1.0209 (3)	0.3382 (2)	0.0211 (6)	
C4	0.5518 (4)	0.8850 (4)	0.3518 (2)	0.0339 (7)	
H4A	0.4639	0.8225	0.2982	0.041*	
C5	0.5904 (4)	0.8402 (4)	0.4447 (2)	0.0360 (7)	
H5A	0.5279	0.7483	0.4534	0.043*	
C6	0.7213 (4)	0.9312 (3)	0.5240 (2)	0.0241 (6)	
C7	0.8153 (4)	1.0642 (4)	0.5093 (3)	0.0423 (9)	
H7A	0.9059	1.1246	0.5616	0.051*	
C8	0.7750 (4)	1.1096 (4)	0.4154 (3)	0.0430 (9)	
H8A	0.8390	1.2002	0.4056	0.052*	
C9	0.8428 (4)	0.9852 (3)	0.7110 (2)	0.0261 (6)	
H9B	0.8012	1.0699	0.7253	0.031*	
H9A	0.9565	1.0214	0.7010	0.031*	
C10	0.8329 (3)	0.9155 (3)	0.8065 (2)	0.0190 (5)	
C11	0.9563 (3)	0.6601 (3)	0.15137 (19)	0.0181 (5)	
C12	1.0010 (4)	0.5810 (4)	0.2341 (2)	0.0243 (6)	
H12B	0.9788	0.4759	0.1988	0.029*	
H12A	1.1177	0.6239	0.2631	0.029*	
C13	0.9586 (3)	0.5444 (3)	0.4075 (2)	0.0206 (5)	
C14	0.8822 (3)	0.5722 (3)	0.4975 (2)	0.0232 (6)	
H14A	0.8028	0.6202	0.4956	0.028*	
C15	1.0760 (3)	0.4714 (3)	0.4092 (2)	0.0230 (6)	
H15A	1.1263	0.4517	0.3484	0.028*	
O12	0.4607 (3)	0.4588 (3)	−0.19590 (18)	0.0376 (5)	
H12D	0.3720	0.3998	−0.1934	0.056*	
H12C	0.4699	0.4849	−0.2530	0.056*	
O13	0.3829 (3)	0.8206 (4)	0.0782 (2)	0.0567 (8)	
H13D	0.2831	0.8057	0.0727	0.085*	
H13C	0.4297	0.9046	0.1180	0.085*	
O14	0.5142 (4)	0.5730 (4)	0.6226 (2)	0.0636 (8)	
H14C	0.5877	0.6523	0.6493	0.095*	
H14D	0.4202	0.5660	0.5976	0.095*	

### Atomic displacement parameters (Å^2^)

**Table:**

	*U*^11^	*U*^22^	*U*^33^	*U*^12^	*U*^13^	*U*^23^
Er1	0.01580 (6)	0.01211 (7)	0.01281 (6)	0.00315 (5)	0.00181 (4)	0.00477 (4)
O1	0.0278 (10)	0.0177 (11)	0.0276 (10)	0.0072 (9)	0.0128 (8)	0.0058 (8)
O2	0.0267 (10)	0.0182 (11)	0.0226 (9)	0.0063 (9)	0.0097 (8)	0.0070 (8)
O3	0.0216 (9)	0.0332 (12)	0.0170 (8)	0.0080 (9)	0.0051 (8)	0.0121 (9)
O4	0.0492 (13)	0.0192 (11)	0.0153 (9)	0.0017 (10)	−0.0075 (9)	0.0049 (8)
O5	0.0204 (9)	0.0196 (10)	0.0136 (8)	0.0039 (8)	−0.0023 (7)	0.0033 (7)
O6	0.0401 (12)	0.0184 (11)	0.0222 (9)	−0.0010 (10)	−0.0054 (9)	0.0071 (9)
O7	0.0259 (10)	0.0254 (11)	0.0203 (9)	0.0089 (9)	0.0066 (8)	0.0128 (8)
O8	0.0283 (10)	0.0270 (12)	0.0234 (9)	0.0126 (9)	0.0078 (8)	0.0140 (9)
O9	0.0357 (11)	0.0370 (13)	0.0191 (9)	0.0213 (11)	0.0086 (8)	0.0164 (9)
O10	0.0248 (10)	0.0174 (11)	0.0422 (11)	0.0076 (9)	−0.0026 (9)	0.0024 (9)
O11	0.0191 (10)	0.0379 (15)	0.0471 (13)	0.0097 (11)	−0.0015 (10)	−0.0118 (11)
C1	0.0199 (12)	0.0210 (15)	0.0123 (11)	0.0076 (11)	0.0018 (10)	0.0057 (10)
C2	0.0336 (15)	0.0248 (16)	0.0195 (12)	0.0145 (14)	0.0110 (12)	0.0110 (12)
C3	0.0233 (13)	0.0291 (16)	0.0135 (11)	0.0096 (13)	0.0060 (10)	0.0079 (11)
C4	0.0394 (18)	0.0275 (18)	0.0242 (14)	−0.0007 (15)	−0.0117 (13)	0.0069 (13)
C5	0.050 (2)	0.0211 (17)	0.0260 (14)	−0.0022 (15)	−0.0099 (14)	0.0078 (13)
C6	0.0339 (15)	0.0229 (16)	0.0145 (11)	0.0078 (13)	0.0009 (11)	0.0059 (11)
C7	0.0423 (19)	0.042 (2)	0.0264 (15)	−0.0108 (17)	−0.0125 (14)	0.0158 (15)
C8	0.0414 (19)	0.041 (2)	0.0341 (17)	−0.0128 (17)	−0.0065 (15)	0.0236 (17)
C9	0.0363 (16)	0.0221 (16)	0.0147 (12)	0.0023 (13)	−0.0018 (11)	0.0056 (11)
C10	0.0196 (13)	0.0215 (15)	0.0164 (11)	0.0066 (12)	0.0010 (10)	0.0060 (11)
C11	0.0234 (13)	0.0131 (13)	0.0145 (11)	0.0014 (11)	−0.0009 (10)	0.0039 (10)
C12	0.0328 (15)	0.0282 (17)	0.0199 (12)	0.0147 (14)	0.0086 (12)	0.0139 (12)
C13	0.0277 (14)	0.0196 (15)	0.0165 (12)	0.0080 (12)	0.0024 (10)	0.0087 (11)
C14	0.0261 (14)	0.0264 (16)	0.0232 (13)	0.0142 (13)	0.0056 (11)	0.0106 (12)
C15	0.0299 (15)	0.0252 (16)	0.0186 (12)	0.0124 (13)	0.0076 (11)	0.0090 (12)
O12	0.0275 (11)	0.0397 (15)	0.0333 (11)	−0.0039 (11)	−0.0022 (9)	0.0064 (11)
O13	0.0216 (12)	0.067 (2)	0.0594 (16)	0.0098 (13)	−0.0010 (11)	−0.0214 (15)
O14	0.0631 (19)	0.052 (2)	0.0439 (15)	−0.0218 (16)	−0.0011 (13)	0.0059 (14)

### Geometric parameters (Å, °)

**Table:**

Er1—O11	2.317 (2)	C1—C2	1.531 (3)
Er1—O2^i^	2.3415 (18)	C2—H2B	0.9700
Er1—O1	2.3437 (19)	C2—H2A	0.9700
Er1—O10	2.368 (2)	C3—C8	1.366 (4)
Er1—O5^ii^	2.381 (2)	C3—C4	1.373 (4)
Er1—O8	2.3996 (18)	C4—C5	1.383 (4)
Er1—O5^iii^	2.4684 (19)	C4—H4A	0.9300
Er1—O7	2.4864 (19)	C5—C6	1.376 (4)
Er1—O6^iii^	2.529 (2)	C5—H5A	0.9300
Er1—C11	2.798 (2)	C6—C7	1.366 (5)
Er1—C10^iii^	2.859 (3)	C7—C8	1.403 (4)
Er1—Er1^i^	3.8505 (13)	C7—H7A	0.9300
O1—C1	1.240 (3)	C8—H8A	0.9300
O2—C1	1.260 (3)	C9—C10	1.505 (3)
O2—Er1^i^	2.3415 (18)	C9—H9B	0.9700
O3—C3	1.392 (3)	C9—H9A	0.9700
O3—C2	1.425 (3)	C10—Er1^iv^	2.859 (3)
O4—C6	1.388 (3)	C11—C12	1.506 (3)
O4—C9	1.417 (3)	C12—H12B	0.9700
O5—C10	1.281 (3)	C12—H12A	0.9700
O5—Er1^ii^	2.381 (2)	C13—C14	1.384 (4)
O5—Er1^iv^	2.4684 (19)	C13—C15	1.388 (4)
O6—C10	1.237 (4)	C14—C15^v^	1.388 (3)
O6—Er1^iv^	2.529 (2)	C14—H14A	0.9300
O7—C11	1.260 (3)	C15—C14^v^	1.388 (3)
O8—C11	1.254 (3)	C15—H15A	0.9300
O9—C13	1.378 (3)	O12—H12D	0.8176
O9—C12	1.411 (3)	O12—H12C	0.8196
O10—H10D	0.8194	O13—H13D	0.8221
O10—H10C	0.8213	O13—H13C	0.8190
O11—H11D	0.8212	O14—H14C	0.8180
O11—H11C	0.8194	O14—H14D	0.8188
			
O11—Er1—O2^i^	140.01 (8)	C10—O6—Er1^iv^	92.30 (16)
O11—Er1—O1	69.84 (8)	C11—O7—Er1	90.52 (15)
O2^i^—Er1—O1	139.47 (7)	C11—O8—Er1	94.74 (14)
O11—Er1—O10	71.00 (8)	C13—O9—C12	114.6 (2)
O2^i^—Er1—O10	84.46 (8)	Er1—O10—H10D	128.3
O1—Er1—O10	135.94 (7)	Er1—O10—H10C	124.8
O11—Er1—O5^ii^	141.78 (7)	H10D—O10—H10C	106.9
O2^i^—Er1—O5^ii^	74.46 (7)	Er1—O11—H11D	130.0
O1—Er1—O5^ii^	71.96 (7)	Er1—O11—H11C	124.3
O10—Er1—O5^ii^	142.81 (7)	H11D—O11—H11C	105.6
O11—Er1—O8	82.71 (8)	O1—C1—O2	127.0 (2)
O2^i^—Er1—O8	125.12 (6)	O1—C1—C2	118.6 (2)
O1—Er1—O8	74.50 (7)	O2—C1—C2	114.3 (2)
O10—Er1—O8	81.33 (8)	O3—C2—C1	113.5 (2)
O5^ii^—Er1—O8	86.19 (7)	O3—C2—H2B	108.9
O11—Er1—O5^iii^	96.38 (8)	C1—C2—H2B	108.9
O2^i^—Er1—O5^iii^	74.53 (6)	O3—C2—H2A	108.9
O1—Er1—O5^iii^	75.37 (6)	C1—C2—H2A	108.9
O10—Er1—O5^iii^	128.65 (7)	H2B—C2—H2A	107.7
O5^ii^—Er1—O5^iii^	74.90 (7)	C8—C3—C4	119.4 (2)
O8—Er1—O5^iii^	148.16 (7)	C8—C3—O3	124.3 (3)
O11—Er1—O7	123.14 (8)	C4—C3—O3	116.3 (3)
O2^i^—Er1—O7	72.14 (6)	C3—C4—C5	120.6 (3)
O1—Er1—O7	119.42 (7)	C3—C4—H4A	119.7
O10—Er1—O7	68.62 (7)	C5—C4—H4A	119.7
O5^ii^—Er1—O7	75.81 (7)	C6—C5—C4	120.1 (3)
O8—Er1—O7	53.28 (6)	C6—C5—H5A	120.0
O5^iii^—Er1—O7	140.25 (6)	C4—C5—H5A	120.0
O11—Er1—O6^iii^	72.43 (8)	C7—C6—C5	119.6 (3)
O2^i^—Er1—O6^iii^	71.57 (7)	C7—C6—O4	124.5 (3)
O1—Er1—O6^iii^	108.94 (7)	C5—C6—O4	115.8 (3)
O10—Er1—O6^iii^	77.05 (8)	C6—C7—C8	120.0 (3)
O5^ii^—Er1—O6^iii^	122.41 (7)	C6—C7—H7A	120.0
O8—Er1—O6^iii^	151.20 (7)	C8—C7—H7A	120.0
O5^iii^—Er1—O6^iii^	52.04 (7)	C3—C8—C7	120.2 (3)
O7—Er1—O6^iii^	131.64 (7)	C3—C8—H8A	119.9
O11—Er1—C11	103.21 (9)	C7—C8—H8A	119.9
O2^i^—Er1—C11	98.81 (7)	O4—C9—C10	109.7 (2)
O1—Er1—C11	97.32 (7)	O4—C9—H9B	109.7
O10—Er1—C11	72.86 (8)	C10—C9—H9B	109.7
O5^ii^—Er1—C11	80.41 (7)	O4—C9—H9A	109.7
O8—Er1—C11	26.52 (7)	C10—C9—H9A	109.7
O5^iii^—Er1—C11	155.31 (7)	H9B—C9—H9A	108.2
O7—Er1—C11	26.77 (7)	O6—C10—O5	121.1 (2)
O6^iii^—Er1—C11	149.19 (7)	O6—C10—C9	122.2 (2)
O11—Er1—C10^iii^	85.37 (9)	O5—C10—C9	116.7 (3)
O2^i^—Er1—C10^iii^	69.14 (7)	O6—C10—Er1^iv^	62.09 (14)
O1—Er1—C10^iii^	94.05 (7)	O5—C10—Er1^iv^	59.45 (13)
O10—Er1—C10^iii^	102.16 (8)	C9—C10—Er1^iv^	170.90 (19)
O5^ii^—Er1—C10^iii^	98.49 (8)	O8—C11—O7	121.4 (2)
O8—Er1—C10^iii^	165.74 (7)	O8—C11—C12	122.0 (2)
O5^iii^—Er1—C10^iii^	26.56 (7)	O7—C11—C12	116.5 (2)
O7—Er1—C10^iii^	140.92 (7)	O8—C11—Er1	58.74 (12)
O6^iii^—Er1—C10^iii^	25.61 (7)	O7—C11—Er1	62.71 (12)
C11—Er1—C10^iii^	167.59 (7)	C12—C11—Er1	178.07 (19)
O11—Er1—Er1^i^	123.86 (7)	O9—C12—C11	110.6 (2)
O2^i^—Er1—Er1^i^	70.32 (5)	O9—C12—H12B	109.5
O1—Er1—Er1^i^	69.30 (5)	C11—C12—H12B	109.5
O10—Er1—Er1^i^	153.26 (5)	O9—C12—H12A	109.5
O5^ii^—Er1—Er1^i^	38.24 (4)	C11—C12—H12A	109.5
O8—Er1—Er1^i^	120.23 (6)	H12B—C12—H12A	108.1
O5^iii^—Er1—Er1^i^	36.66 (5)	O9—C13—C14	115.1 (2)
O7—Er1—Er1^i^	109.95 (5)	O9—C13—C15	124.5 (2)
O6^iii^—Er1—Er1^i^	86.39 (6)	C14—C13—C15	120.3 (2)
C11—Er1—Er1^i^	118.65 (6)	C13—C14—C15^v^	120.2 (2)
C10^iii^—Er1—Er1^i^	61.18 (7)	C13—C14—H14A	119.9
C1—O1—Er1	137.57 (17)	C15^v^—C14—H14A	119.9
C1—O2—Er1^i^	135.37 (17)	C13—C15—C14^v^	119.5 (2)
C3—O3—C2	119.0 (2)	C13—C15—H15A	120.2
C6—O4—C9	116.5 (2)	C14^v^—C15—H15A	120.2
C10—O5—Er1^ii^	146.22 (17)	H12D—O12—H12C	116.6
C10—O5—Er1^iv^	93.99 (16)	H13D—O13—H13C	108.2
Er1^ii^—O5—Er1^iv^	105.10 (7)	H14C—O14—H14D	124.5

Symmetry codes: (i) −*x*+2, −*y*+2, −*z*; (ii) −*x*+2, −*y*+2, −*z*+1; (iii) *x*, *y*, *z*−1; (iv) *x*, *y*, *z*+1; (v) −*x*+2, −*y*+1, −*z*+1.

### Hydrogen-bond geometry (Å, °)

**Table:**

*D*—H···*A*	*D*—H	H···*A*	*D*···*A*	*D*—H···*A*
O10—H10D···O7^vi^	0.82	2.09	2.880 (4)	163
O10—H10C···O12	0.82	1.97	2.732 (4)	154
O11—H11D···O13	0.82	1.82	2.634 (4)	173
O11—H11C···O12	0.82	1.92	2.709 (4)	161
O12—H12D···O8^vii^	0.82	2.00	2.798 (4)	167
O12—H12C···O14^iii^	0.82	1.97	2.780 (4)	172
O13—H13D···O7^viii^	0.82	2.12	2.872 (4)	151
O13—H13C···O3	0.82	2.03	2.804 (4)	157
O14—H14C···O6	0.82	2.17	2.874 (4)	144

Symmetry codes: (vi) −*x*+2, −*y*+1, −*z*; (vii) −*x*+1, −*y*+1, −*z*; (iii) *x*, *y*, *z*−1; (viii) *x*−1, *y*, *z*.
